# Living bioethics, theories and children’s consent to heart surgery

**DOI:** 10.1177/14777509221091086

**Published:** 2022-04-07

**Authors:** Priscilla Alderson, Deborah Bowman, Joe Brierley, Nathalie Dedieu, Martin J Elliott, Jonathan Montgomery, Hugo Wellesley

**Affiliations:** 1 Social Research Institute, University College London, London, UK; 2 Medical Ethics, 4915 St George’s Hospital, London, UK; 3 4956 Great Ormond Street Children’s Hospital, London, UK; 4 Cardiothoracic Surgery, University College London, London, UK; 5 Faculty of Law, University College London, London, UK

**Keywords:** Philosophical aspects, bioethics and medical ethics, clinical ethics, minors, care for specific groups, health care quality, health care, informed consent, professional ethics in medicine, professional ethics in nursing

## Abstract

**Background:**

This analysis is about practical living bioethics and how law, ethics and sociology understand and respect children’s consent to, or refusal of, elective heart surgery. Analysis of underlying theories and influences will contrast legalistic bioethics with living bioethics. In-depth philosophical analysis compares social science traditions of positivism, interpretivism, critical theory and functionalism and applies them to bioethics and childhood, to examine how living bioethics may be encouraged or discouraged. Illustrative examples are drawn from research interviews and observations in two London paediatric cardiac units. This paper is one of a series on how the multidisciplinary cardiac team members all contribute to the complex mosaic of care when preparing and supporting families’ informed consent to surgery.

**Results:**

The living bioethics of justice, care and respect for children and their consent depends on theories and practices, contexts and relationships. These can all be undermined by unseen influences: the history of adult-centric ethics; developmental psychology theories; legal and financial pressures that require consent to be defined as an adult contract; management systems and daily routines in healthcare that can intimidate families and staff; social inequalities. Mainstream theories in the clinical ethics literature markedly differ from the living bioethics in clinical practices.

**Conclusion:**

We aim to contribute to raising standards of respectful paediatric bioethics and to showing the relevance of virtue and feminist ethics, childhood studies and children’s rights.

## Introduction

Consent to surgery has been understood in the past as an event which includes signing a form, within individual surgeon-patient relationships.^1^ However, our recent study of children’s elective heart surgery observed how multidisciplinary teams prepare, inform and support families through the extended consent process.^2^ This involves cardiologists, surgeons, anaesthetists, nurses, play specialists, psychologists, social workers, chaplains and others. Bioethicists and lawyers also help to raise standards and respect for informed and voluntary consent through their extensive literature, formal inquiries, judicial decisions, professional guidelines, study days and degree courses, which many healthcare professionals attend, and via their work in clinical ethics committees. This paper examines the contributions from law and ethics. A companion paper considers living bioethics and clinical ethics committees.^3^

Legalistic bioethics theorises children as pre-competent, unable to make rational informed decisions, or to give valid consent to surgery, or refuse it. Legalistic bioethics will be contrasted with the living bioethics in the actual practice of the ethical principles of care and respect for children’s consent or refusal. Practice was researched in paediatric cardiology departments in two London hospitals. The research included: literature reviews; observations in clinics, wards and medical meetings, October 2019 to February 2020; audio-recorded and transcribed interviews with 45 healthcare professionals and related experts; discussions with a multi-disciplinary advisory group; and thematic qualitative data analysis. Contact with participants was by phone and online only, from March 2020 to April 2021, hence data collecting was severely reduced by the Covid-19 pandemic. Interviews with children aged 6–15 years, and their parents, about their elective heart surgery are reported in other papers.^1^

This paper analyses how different traditions and paradigms in bioethics support different views about ethics and children and their consent. The paper draws on a similar analysis of traditions in sociology, which is summarised in the next section, to see how that can inform analysis of bioethics.

## Four traditions in social science

Sociology divides into three main traditions or paradigms (world views): positivism, hermeneutics and critical theory.^4^ There is also the paradigm of functionalism which is a political position rather than a research tradition, and can influence any method.

*Positivism,* in the Enlightenment tradition of Hume, Compte and Durkheim is now by far the most highly funded and trusted version of social science. Following natural science epitomised in physics, positivist social scientists search for predictable, measurable laws and systems. Their surveys and randomised controlled trials of numerous individuals study patterns in their beliefs and behaviours analysed by variables such as class, race, age or gender. The Scientific Advisory Group for Emergencies, SAGE, which has advised the UK Government on COVID-19, relies on positivists such as behavioural scientists. Predictive positivism works extremely well in the natural sciences, for example, in trials of cancer chemotherapies. Yet positivism is less effective in social research about complex unpredictable human beings. SAGE predicted that people would not tolerate restrictions imposed during the first lockdown, in 2020.^5^ But SAGE was shown to underrate individuals’ mixed concerns not only for their own interests and freedoms but also for the protection of others especially the vulnerable, and for solidarity and social justice.

*Hermeneutics*, following Kant, Weber and Heidegger and unlike positivism, recognises great differences between the natural and social sciences. Individuals are observed and interviewed, usually in small, detailed samples, about their diverse personal views and experiences, often reported in case studies.

*Critical theory,* informed by Marx, Adorno, Gramsci and Habermas, aims for major changes in how decisions and resources can be controlled and shared more equally. ‘Critical’ denotes Marx’s aim to change as well as to interpret the world. Researchers of poverty, post-colonialism and disability, for instance, campaign for justice, inclusion and flourishing in new ways of everyone living together.

*Functionalism,* influenced by Durkheim, Talcott Parsons and Merton, is the belief that everything generally functions for the greater good of all, in contrast to critical theory. A conservative consensus of shared norms and values is assumed. The first three paradigms influence research design and methods, whereas functionalism is an often unacknowledged, though dominant, political view usually supported by positivists.

Each tradition has different views of ethics.

*Positivists* aim for value-free objectivity,^6^ attempting to ignore the ethics latent in health research. Rather than make recommendations, they hope self-evident policy conclusions will flow from the scientific evidence. However, after decades of their evidence being ignored by policy makers, positivists such as Michael Marmot on public health^7^ or David Attenborough on climate change are becoming more overtly critical, moral and political.

*Interpretive or hermeneutic* researchers also prefer value-neutrality and aim to set aside their own views and to understand the views or positions of others. Yet their non-judgmental reports about unjust or abusive behaviours and systems can seem relativist or amoral (Sayer).

*Critical theorists* contend that all social life is imbued with moral meaning.^8^ We are evaluative beings who flourish and suffer, so we cannot separate social facts from values. Imagine a doctor tells you your blood pressure reading. You ask what you should do, and the doctor replies, ‘Well, I can’t tell you because that would be a value judgment and it would compromise my objectivity, and I can’t advise you what to do because you can’t derive an ought from an is’. ^9^ Critical theorists take objectivity to mean being fair, open-minded and rigorous but not being value-free. Researchers who ignore or deny their values are likely to be subconsciously biased by them, as the long history of sexist and racist social and medical research shows. Critical realists critically debate explicit and conflicting values and interests.

*Functionalists* tend to assume they are value-free when they support seemingly unquestioned taken-for-granted norms and utilitarian calculations. Functionalists support stable structures of dominant and subordinate groups, such as the ruling and working classes, or adults and children who must be disciplined and educated until they become adults. Similarly, interpretivists tend not to question inequalities but study how they shape identities and social symbolism. Like positivism, functionalism is more or less generally assumed to be the usual neutral factual position. Yet functionalists take sides, and support powerful groups, when they believe problem events and people need to be corrected and reformed to aid efficient societies, just as healthcare professionals treat illness and restore health. Social positivists tend to be functionalist when they attribute problems to individuals’ behaviours, and they evaluate remedies to alter them through controlled trials, such as support groups to reduce obesity rates. This ‘bad apples in the benign barrel’ worldview is challenged by critical theorists who are concerned with ‘bad barrels’. And instead of researching effects, such as obesity, they look ‘upstream’ for prior causes of these ills, such as the obesogenic food, retail and leisure industries.^10^ Blame and the hope for real change then shift from individuals alone to political and economic systems. But these are topics which ‘objective’ researchers avoid as ‘biased’ believing that functionalist research is neutral, value-free and non-political. Critical theorists, however, consider no research tradition can escape bias and no ‘objectivity’ be fully attained, so all researchers need to be critically aware of their own value-systems.

## The four traditions and bioethics

How might the traditions in social science relate to bioethics? This section considers how: *positivists* prefer to apply clear abstract general laws and principles and evidence-based calculations; *interpretivists* favour complex analysis of diverse individual understandings; *critical theorists* use both approaches but also advocate greater awareness of power to redress inequalities; and *functionalists* accept hierarchies and inequalities, when they appear to support stable efficient societies.

The social sciences involve theories and methods of ontology and epistemology, which concern what exists and how we can know what we know. Yet theories also involve paradigms, world views with often hidden moral and political implications. Similarly, bioethics involves different paradigms, such as deontology and utilitarianism that address methods of analysis and moral worldviews. During the mid-twentieth century, bioethicists constructed bioethics systems that tended to be intellectual, abstract and impersonal.^11^ They were influenced by the US civil rights movement, which prompted external scrutiny based on formal documentation and legalistic norms.^12^ This influenced bioethics in the UK to apply forms of accountability based on the utilitarian positivist measurement-audit, managerial control and suspicion of claims of professional values.^13,14^ Philosophical bioethicists became the dominant adjudicators of medical morality when they made universalist claims to be able to rise above their personal values and cultural constraints.^15^ Deontology offers methods of analysis that echo positivism’s definitive and predictable laws or principles. Utilitarianism relies on positivist evidence and methods of measuring and evaluation.

Many doctors may be attracted to formal bioethics by its positivist and medical-like concern to identify or diagnose the problem in individual cases, agree a consensus informed by universal laws, prescribe a remedy, and hope to improve the prognosis. This is not necessarily a personal preference, but fits methods emphasised in medical training and practice. Medicine, law and bioethics all draw on revered ancient European traditions and precedents such as those influenced by Hippocrates, Cicero or Aristotle.

Although dealing with values, *functionalist* legalistic bioethics may be seen as neutral or value-free when administering impersonal moral principles and current norms or utilitarian calculations that assume dominant priorities. Yet legalistic bioethics supports present power structures,^16^ such as laws that exclude legal minors’ decisions. It tends to assume the authority of law,^17,18^ disregarding the contingencies of law-making processes, the claimed foundations of legal jurisdiction and techniques that maintain its dominance over bioethics.^19,20^

*Interpretivists and critical theorists* challenge positivists’ claims that their work is neutral and universal. Critical theorists critically question and challenge claims about ‘justice’, ‘rights’ and ‘values’.^21^ Feminists have shown how mainstream ethics neglects gender differences, emotions and care.^22^ Antiracists envisage all bioethicists ‘dismantling their privilege and advocating for a more inclusive and equitable bioethical discourse’.^23^ Some social scientists hope to enrich bioethics by expanding its social and political dimensions.^24,25^ Some contend that bioethics is too top-down, and takes too little account of social evidence and individuals’ needs, so that bioethicists may unwittingly support widespread prejudices and systems such as individualism, promoting autonomy over community.^26^

Other social scientists want bioethicists and ethics committees to attend more to their real messy interpersonal decision-making, unlike the presumed impersonal, de-socialised and mechanical bioethics model.^27^ Practitioners highlight ‘the obstacles for moral action instead of the conceptual analysis of some supposedly universal and unchanging norms’.^28^ This would involve moving from narrowly-framed analytical rational thought towards more self-aware dialogue through critical questioning of taken-for-granted theories and power relations.^29^ Bioethicists would then be more empirically rooted, theory challenging, politely sceptical and critically aware of their own assumptions.^30^ Communitarian ethicists want more equality, less dominance by powerful professions, more attention to luck and misfortunes beyond our limited control, with an ethic of humility and solidarity that is more affirming of the dignity of lower status people.^31^

Mainstream analytical ethicists tend to separate theory from practice; analysing, knowing and talking about ethics versus the being and doing of living ethics.^32,33^ To concentrate on reasoned decisions, and to believe in the Kantian tradition that emotions undermine rationality,^34^ risks compromising bioethicists’ and lawyers’ own empathy. If children are assumed to have no legal rights to be informed or involved in decisions about their surgery, interventions may be enforced on resisting children, ‘for their own good’.^35^ Practitioners recently reported that children are restrained during procedures ‘quite often (48%) or very often (33%)’ in what would be illegal assault on an adult patient.^36^ This can violate the ethical principles of: respect for the person’s autonomy; beneficence that acts in the patient’s best long-term interests (discussed later); non-maleficence and not doing harm; justice and treating all patients with equal care and respect.^37^ Living ethics involves actual caring respectful practice as much as theory. Legalistic bioethicists seek to abstract and apply general constructs and principles to each case they examine. However, healthcare practitioners constantly work with living ethics, responding to individual patients’ needs and fears, suffering or complaints.^38^ Like critical theory, today’s virtue ethics aims to be reflexive, aware of power and personal beliefs, and it connects theory to practice.^39^

Aristotle considered that ‘moral excellence [i.e. virtue] comes about as a result of habit’ and that ‘we become just by doing just acts, temperate by doing temperate acts, brave by doing brave acts’.^40^ Living ethics can be refined and strengthened through the practice of virtuous habits and enduring character traits, rather than through single actions. (This does raise questions about how we can begin to act kindly or bravely without first being kind or brave. It could also misleading imply that young children cannot be kind or brave.) In virtue ethics there is less deontological formal emphasis on duties, and more concern with pondering and enacting the good life and the good person.^41^ These are informed by Aristotle’s 18 virtues, each a golden mean between extremes: courage avoids either fear or recklessness, and modesty avoids either shame or shamelessness. Virtue ethics has influenced capability theory in the living bioethics values of theory-practice consistency (enacting our beliefs), practical moral wisdom, the well-lived life and flourishing. ^42,43^^,^ In critical realism, there is similar concern to be actively virtuous, beyond superficial discussion of virtues. ‘Each is true to, of, in and for themselves and every other (including future generations and other species) subject to the constraints imposed by nature.’^44^

Yet there are debated uncertainties. Should virtuous persons be trusted, and granted the discretion and power to decide on other people’s moral questions? How can disagreements about virtuous decisions be resolved? Virtue ethics has been sexist and racist in the past, and it risks being relativist when prized virtues vary between different cultures. How rigorously should virtues and ensuing decisions be explained and accounted for? Nevertheless, striving to be a good doctor or nurse highlights virtues in each personal relationship.^45^ The clinician listens, respects the views of others and keeps their secrets, as well as telling the truth, helping not harming, admitting error, trying to decide what is right during each ethical difficulty, and searching for common values in discussions with patients, through the virtuous habits of compassion, prudence, altruism, trustworthiness and humility. During our research interviews, a children’s heart surgeon and clinical ethics committee member trained with two surgeons ‘who hated each other’ and then with two who respected one another.

There was a trickledown effect in the department. Everyone held their tails up, and there I learnt how to conduct my professional life, and I thought, that is what I want to emulate in my conduct [and in teaching my trainees] how to behave to everyone around them. [Ethics] is a lifestyle almost a faith if you like.

Virtuous individuals need to be supported by effective systems, such as a management framework that works to reduce and prevent conflict between clinical staff and families, with staff training and support and teamwork and mediation.^46^ The research interviews found that the key clinician to manage a child’s refusal of elective surgery is the anaesthetist. A zero-restraint ethos supports all the anaesthetists to refuse to force the mask or cannula onto children from around 4 years of age onwards. If they firmly resist the anaesthetic, non-emergency heart surgery may be cancelled. Play specialists, nurses and psychologists work with these children until they are ready to understand and accept the surgery. The cardiac anaesthetists value the regular multi-disciplinary clinical team meetings. These critically review problem cases and ensure that patient-centred policies are developed, agreed and adhered to by all members of the team.

The next section considers differing approaches to childhood and to ethics.

## Four traditions, childhood and ethics

Until the 1990s, social research about childhood was dominated by positivist Piagetian biology-based development theory, which climbs from an imagined zero at birth up to mature adulthood.^47^ Just as feminism respects women, the ‘new’ childhood studies are concerned with children’s views, experiences and rights.^48^ Concepts of ‘childhood’ are criticised as often oppressive social constructions that justify adults’ power and underestimate children’s capacities. Numerous international studies show how children’s competence depends far more on their social experiences than on their biological age.^49^

There is not space here to review the extensive bioethics literature on children. Instead, a typical example that illustrates the broadly positivist functionalist approach will be considered.^50^ The authors assume contradictions between interests and rights, between ensuring that ‘the best interests of the child shall be a primary consideration’ versus the child’s right, when ‘capable of forming his or her own views … to express those views freely in all matters affecting the child, the views of the child being given due weight in accordance with the age and maturity of the child.’^51^ They consider that ‘best interests’ are understood by adults but liable to be misunderstood by immature potentially self-harming children. They review law court cases in England and Norway to identify problems and good standards. The paper adopts positivist and functionalist traditions: the search for generally respected standards, precedents and procedures; facts and evidence provided by experts; tests and evaluations of children’s (im)maturity and (in)ability; dualisms to clarify analysis such as child/adult, competent/incompetent, interests/rights. The five English court cases, cited in this and numerous other papers, involve mental illness, anorexia nervosa, Jehovah’s Witness young people who refused blood transfusion, and refusal of a heart transplant.^52^ All the cases involve complex major decisions about treatment for serious, often life-threatening conditions, and involve older children who disagree with the professionals’ views of their best interests. The authors deem children to be incompetent if, like the child in the heart transplant case, they are ‘overwhelmed’, and if they show ‘an emotional reaction’, ‘ignorance or a poor understanding’ and ‘over-reliance on the judgements of others’. Competence is seen to involve ‘an ability to come to terms, presumably dispassionately and calmly, with a life-threatening situation, [and] a broad experience of life’.^53^ The young people cited in the paper all appear to be unreasonable, so that the more carefully procedures for consulting children and assessing their maturity are set out, as in the paper, the less worthwhile it appears to be to consult them. Such efforts appear to demonstrate children’s general incompetence and need for wise overriding adult protection. The paper is typical of many bioethics papers about children in that it does not examine how the children’s detailed biographies and backgrounds might explain their reasoning. It disregards how the adults might have informed or not informed the children concerned, and it ignores younger children. Also avoided, with children’s decisions in controversial cases, is how the adults concerned might disagree. Generally in healthcare, controversial and under-researched treatments are not so uncommon.^54^ The largest omission is the great majority of children who make reasonable decisions with which the adults agree. General conclusions for ethics and policy based on unusual controversial legal cases can be unrealistic. Positivist bioethics is oppositional in its emphasis on children jeopardising their adult future and disagreeing with adults. Patriarchal philosophy and law are used to justify parental power as the remedy for seemingly inevitable deficits in legal minors’ agency and choices in a range of clinical cases.^55^

In the contrasting interpretive *Gillick* standard in English law, the consent of minors aged under-16 is legally valid if they ‘understand fully what is proposed’, and ‘can make a wise choice’ in their best interests.^56^ No minimum age is specified. ‘The parental right yields to the child’s right to make his [*sic*] own decisions when he reaches a sufficient understanding and intelligence to be capable of making up his own mind on the matter requiring decision’, and parents’ rights to decide ‘terminate’ when their child become *Gillick* competent. Yet in most cases of surgery there is consensus. Children prefer to share decision-making with their healthcare professionals and family, just as many adult patients do, although it is still vital in these cases to respect children’s informed willing consent, and prevent avoidable fear, ignorance and coercion.

Legalistic bioethics theory differs from much everyday ethical practice. A common generalisation in the law and ethics literature is that children ‘cannot refuse’. Yet, first, children do refuse. Second, children’s refusal is necessary for their wellbeing and even survival. Babies try to resist being put into an over-hot bath, and young children learn to say ‘no’ to strangers. Third, ‘children cannot refuse’ is shorthand for ‘children’s refusal of medical treatment cannot be accepted in law’. However, in the UK, both pre-competent and *Gillick* competent minors ‘successfully refuse treatment in the NHS countless times every day. They decline [and spit out] oral medications, ignore the advice of physiotherapists and refuse point blank to be anaesthetised for operations that could improve the quality of their lives’.^57^ The courts cannot enforce treatment, but only authorise doctors to provide it. And doctors are warned to use force, such as restraint, solely as a last resort.^58^ The zero-coercion ethos about heart surgery was mentioned earlier. With heart transplants, the shortage of donor hearts, and the need for life-long follow-up active cooperation with daily immuno-suppression treatment lead clinicians to select patients with great care and to respect children’s reluctance and refusal. Some bioethicists criticise researchers who advocate more respect for children, for example by saying they ‘offer analyses of power without any evaluation of its exercise’.^59^ Yet this ignores empirical research on the benefits of respecting and working with children and the harms of not doing so.^60,61^

Clinicians and researchers do not intend to intimidate children, but formal tests of competence, and research with closed questions requiring correct answers may be perceived and experienced as intimidating and mistrustful by the person being questioned. However, in interpretive research and interactive clinical practice, the adult and child may together see how far they can share information and support and discover not what level the child is at but what the child wants and needs and might be willing and able to know. Nurses and play specialists routinely do this before heart surgery through talking, play activities and materials, interactive body language and humour, all showing adults’ empathy with the child. A play specialist described weekly sessions with a very resistant 12-year-old with learning difficulties who at first refused to take off her coat or sit on the bed. Two play specialists reversed the power relations when they lay on the bed and invited the girl, with much laughter, to press the button that raised and lowered the bed. When later she refused to cooperate with the X-ray, ‘So we did silly [games] on a monkey doll first [laughter]. We said, “All you have to do is hug the X-ray”. We managed to unzip her coat and as she hugged the X-ray, the woman took the picture’.

Consent to surgery is intended to protect patients from unwanted or unhelpful treatment. Consent also protects healthcare professionals and hospitals from complaint and litigation when, by signing the consent form and provided they have been adequately informed, patients accept responsibility for recorded risks and harms. These are central concerns at positivist institutional, legal and financial levels.^62,63^ The concerns discourage lawyers, ethicists and researchers from expecting children aged under-12 to be able to consent.^64^ In contrast, practitioners’ living bioethics and daily interactions encourage them to respect young children. Children assessed for an elective heart transplant stayed for days in hospital having tests and discussions. A nurse manager said,

… a little boy who was about six … really understood the concept of it and he knew exactly what it meant. And in the same way, there was a little girl of a similar age and she definitely didn’t want it. And you can understand the conflict that creates within a family because the parents don’t want to lose their child, and yet in certain areas of medicine the compliance of the child is so pivotal that if they are not on board with it then you’ve wasted a huge resource.

Most of the healthcare professionals interviewed took very seriously young children’s need to be informed. Like some of her colleagues, one cardiologist spoke of starting to explain the meaning and purpose of heart surgery, sometimes against the parents’ wishes, to children aged from 3 years. She wanted to protect her patients from being bewildered, anxious and distressed.

They [the children] are very perceptive and they are smart. They do notice things. I think it's difficult to build trust and it's difficult for them if the people they trust, like their parents or their doctor, are not honest with them and not completely open with them. I don't think that's right.

She added, ‘I don’t think there’s an [lower] age limit. I think you should always talk to the children and tell them that something is going to happen’. She was observed to do this with babies, soothing them by gently telling them how she was going to examine them. She described a 6-year-old who was not informed, and after surgery ‘he was so scared and angry … Psychologically, I think this is terrible for the child’.

Trust is mutual, supported by the virtues mentioned earlier, when healthcare professionals’ knowledge and power serve their patients and do not dominate them, enhanced by mutual trust and respect between all members of the paediatric cardiac team. For example, doctors praised play specialists who enable resisting children to overcome their fears and cooperate with treatment.

Interpretivism includes understanding or standing in the position of others (*Verstehen*) and reinterprets the positivist criteria for maturity listed previously.^47^ Children and adults have ‘an emotional reaction’ and can feel ‘overwhelmed’ by sudden news that they need major treatment. These are not weaknesses but are integral to understanding. They journey through moral emotions, from initial rejection, fear and doubt, through uncertain reflection on the risks of treatment and of the untreated condition, towards hope, trust in the clinical team, courage, confidence and commitment. The journey may take moments or years, but hesitancy does not mean incompetence, and neither does a seeming ‘over-reliance on the judgements of others’, because parents too have to trust doctors’ expert advice. Many children ‘come to terms … with a life-threatening situation’, but if anyone does so ‘dispassionately and calmly’ there would be doubt that they really understand the risks and suffering they face. The criterion of ‘a broad experience of life’ can unfairly exclude children, especially those with chronic illness who live confined lives. Informed consent to major surgery involves deep rather than broad knowledge, gained by many of these children, including many who have learning difficulties, through intensely relevant embodied experience. Attempts to assess a child’s general competence unrelated to the heart problem ignores how these children can often be mature and highly informed about their treatment, but may seem immature in some other aspects of life. Their knowledge cannot be adequately assessed or researched through positivist closed standardised questions, but requires open questions that help children to speak on their own terms.

The most detailed example is the book by Hannah Jones and her mother Kirsty, a former intensive care and transplant nurse.^65^ They explain in over 300 pages why Hannah waited for years before she consented to a heart transplant when she was 14. Her parents always respected her, from when she was 4 years old and needed six cycles of chemotherapy to treat her leukaemia. The chemotherapy damaged her heart so severely that they had to choose whether to consent to a third and final round of chemotherapy ‘to kill the bugs in your blood’, or to refuse it to keep ‘your poorly heart’ safe. When asked for her view, Hannah said she would have to think about the decision. She longed to end the painful treatment and go home. However, she told the doctor, ‘I have decided that I will have some more medicine’. Her parents believed they could not and should not enforce chemotherapy against her wishes. Interpretive research and critical theory both examine how control over decisions, resources and time are shared, or not shared, between adults and children.

## Conclusion: Ways forward in living bioethics concerned with children

The living ethics of justice, care and respect for children and their consent depends on theory and practice, contexts and relationships, systems and individuals. Living ethics can be undermined by unseen influences, referred to earlier. These range from: the history of ethics with its emphasis on an imagined emotion-free adult-centric rationality; misleading developmental psychology; legal and financial pressures on the need for consent to be an adult contract; management systems and daily routines in healthcare that can intimidate and cause stress and anxiety for families and staff; social inequalities of class, race, gender and age. Unless these are actively attended to, their subconscious and practical effects are powerful.

Each approach to ethics has strengths and weaknesses. Positivist concern with evidence and reason is vital for informed consent. Interpretive approaches are needed to help everyone to understand the relevant knowledge and the range of views concerned more widely and deeply, and to respect the moral emotions of trust and voluntariness. Yet positivism can be impersonal and interpretivism can be vague. Functionalist concern for utility and maximising welfare is valuable, although the tendency to attribute problems to individuals and groups rather than to social structures and systems can be unjust and ineffective.

A broader more critical and holistic approach is needed. We hope that this paper will contribute to raising standards of respectful bioethics and to serving child patients in three ways, by:

1. publicising the respectful living ethics in paediatric clinical care in the hospitals studied which is widely practised elsewhere;
2. suggesting that positivist, interpretive, functionalist and critical approaches could be more closely combined in bioethics analysis, guidance and practice, using their complementary strengths; and
3. showing the relevance of virtue ethics, the feminist ethics of care, childhood studies and research on children’s rights and competence, to the theory and practice of clinical ethics.
